# Correlation between follicle diameter and pre-ovulatory metabolic profile in *Bos grunniens*

**DOI:** 10.3389/fvets.2025.1498703

**Published:** 2025-01-28

**Authors:** Jiuru Yan, Yuxin Fu, Lan Lan, Huizhu Zhang, Ling Huang, Yaying Wang, Xianrong Xiong, Shi Yin, Jian Li, Honghong He

**Affiliations:** ^1^Key Laboratory of Qinghai-Tibetan Plateau Animal Genetic Resource Reservation and Utilization, Ministry of Education, Chengdu, China; ^2^College of Animal and Veterinary Sciences, Southwest Minzu University, Chengdu, China; ^3^Key Laboratory of Animal Medicine, Southwest Minzu University, Chengdu, China; ^4^Animal Husbandry Science Institute of Ganzi Tibetan Autonomous Prefecture, Kangding, China

**Keywords:** follicular development, metabolomics, granulosa cells, ovary, *Bos grunniens*

## Abstract

**Introduction:**

In this study, we investigated the metabolic profiles of yak (*Bos grunniens*) follicles during the development period from the perspective of metabolomics, aiming to screen out the differential metabolites of yak follicles in different sizes and potential pathways during yak follicle development and to provide a basis for the study of follicle development and developmental mechanisms in the further stage of development.

**Methods:**

A total of 20 four-year-old female yaks were selected, and follicles of different sizes were collected after slaughter and divided into d < 3 mm, 3–6 mm and d > 6 mm. The follicular fluid was collected, 6 replicates per group and subjected to LC–MS assay, combined with multidimensional and unidimensional statistical analyses to screen the differential metabolites between follicles of different sizes. Differential metabolites enriched KEGG pathways to screen the pathways that might be related to follicle development.

**Results:**

We found that most of the metabolites were mainly enriched in amino acid metabolism pathways, energy metabolism pathways and other pathways of cofactor synthesis, and that during the development of the small follicle to the large follicle, 2-Lysophosphatidylcholine, PC (17:0/0:0), PC (16:0/0:0), and LysoPC (18:0/0:0) were down-regulated; Dioctyl succinate, P-Coumaraldehyde, ISOPRENE, L-Isoleucine, Dioctyl succinate up-regulated.

**Conclusion:**

These results suggest that amino acid metabolism, the production of steroid hormones and their metabolites, and the metabolic activity of granulosa cells play important roles in follicle development. The results provide a theoretical basis for further exploration of follicular development in yak.

## 1 Introduction

The yak is the traditional livestock of the Chinese highlands. However, the reproductive efficiency of yaks is low, usually one or two births every 2 years or two births every 3 years. The important reason that affects reproductive efficiency is the number of follicles ovulated in the ovary. Differentiate and release mature oocytes for fertilization and the successful continuation of the species (1). There are numerous follicles in the ovary, the follicle is the functional unit of the ovary, consisting of an oocyte that is surrounded by granulosa cells and theca cells (2), granulosa cell metabolism is essential for the maintenance of normal fertility, and metabolic changes can regulate follicular and oocyte development by modulating granulosa cell energy metabolism, proliferation, autophagy, and the production of steroid hormones and their metabolites (3). Whereas the glycolytic pathway is the main source of energy for follicular granulosa cells as well as for oocytes, which have a limited capacity to utilize glucose, pyruvate and lactate are the main sources of energy metabolism for granulosa cells, and thus lipid metabolism in granulosa cells is critical during oocyte maturation (4). And oocyte growth depends on the function of follicular cells such as granulosa and theca cells (5). The follicular fluid is an essential microenvironment for oocyte survival, which contains cytokines, hormones and other mediators associated with oocyte maturation and ovarian function. The composition and the levels of these mediators are associated with oocyte fertilization and follicle maturation (6, 7). Not only that, but also the regulatory mechanism of follicle formation, activation and growth is closely related to the complex interaction between local paracrine/autocrine factors and follicular endocrine hormones (8). Folliculogenesis begins with the activation of primordial follicles and their transition to primary follicles, during each estrous cycle or interovulatory interval (IOI). Ovulation occurs from wave 2 in the two-wave IOI and wave 3 in the three-wave IOI (9). A adominant follicle develops during each wave through a process of diameter deviation and selection (10). Only a few dominant follicles can mature to ovulation, while most follicles eventually undergo follicular atresia.

The metabolic factors in the follicular fluid also play a crucial role in the process of follicular development and atresia, metabolites are building blocks of cellular function. Many of the dynamic processes that occur during oocyte growth and maturation require energy, oocytes and follicles can use multisubstances to support their energetic and anabolic needs. These species are involved in enzyme catalyzed chemical reactions and are essential for cellular function (11). Such as NADH/NAD+ ratio, which has a significant impact on energy. NADH is naturally autofluorescent, and previous studies have established that fluorescence measurements of free and bound forms of NADH can be utilized to deduce cellular metabolism production, cell survival, proliferation, longevity, and aging (12–14). These studies suggest that these factors also play a very important role in the process of follicular development.

Metabolites are the result of both biological and environmental factors providing great potential to connect knowledge of genotype and phenotype (15). In order to detect metabolites in cells and reflect the true functional state of biological systems, Nicholson first proposed the concept of metabolomics in 1999. With the development of metabolomics, detection technology has been improved to detect more and more metabolites accurately. Metabolomics has a variety of separation and detection techniques including liquid chromatography (LC), gas chromatography (GC), capillary electrophoresis (CE), mass spectrometry (MS) and nuclear magnetic resonance (NMR). One of the most commonly used detection techniques is liquid chromatography-mass spectrometry (LC–MS). The small molecule metabolites in specific biological samples are quantitatively and qualitatively analyzed using a variety of analytical methods. It can dynamically monitor the changes of metabolites in the body caused by internal and external factors. Metabolomics is a downstream complement to genomics, transcriptomics and proteomics and is the gold standard for analysing metabolic differences (16), and comprehensively and systematically study the metabolic characteristics of organisms (17). In this study, yak follicles were classified according to their diameter. Different sizes of metabolites within the follicles were detected by LC–MS to explore the differences of metabolites in varying sizes of yak follicles, and to find the potential pathway of follicle development, providing a theoretical basis for improving the reproductive performance.

## 2 Materials and methods

### 2.1 Sample collection

All experimental animals were obtained, retained and slaughtered in accordance with local laws and regulations in Sichuan Province, China. Thirty healthy 4-year-old yaks in oestrus were randomly selected for slaughter. Ovaries were isolated, and the fat and connective tissue around the ovaries were cut off with sterile scissors. A total of 60 ovaries were collected and carefully washed with saline to prevent follicle rupture. Ovaries were placed in 37°C saline with penicillin and streptomycin added and transported back to the laboratory within 6 h. Ovaries were washed again with saline at 37°C and processed on an ultraclean table. On average, there were 7 follicles per ovary. Follicles of different sizes were measured and categorized into d < 3 mm, 3–6 mm and d > 6 mm in diameter, with six replicates in each group, and the follicular fluid was extracted with a syringe and placed in freezing tubes and preserved in liquid nitrogen to prepare for subsequent metabolomics assays.

Taken 150 μL sample to 1.5 mL and add 600 μL extract [methanol/acetonitrile = 1:1:Acetonitrile =1:1 (v:v)], including four internal standards [L-2-chlorophenylalanine (0.02 mg/mL), etc.], vortex mix for 30 s, sonicate at low temperature for 30 min (5°C, 40 HZ) and leave the sample at −20°C for 30 min. Centrifuge for 15 min (13,000 g, 4°C), remove the supernatant and dry with nitrogen; add 120 μL complex solution (acetonitrile: water = 1: 1) to redissolve; vortex mixing for 30 s, low temperature ultrasonic extraction for 5 min (5°C, 40 KHz); centrifuge for 10 min (13,000 g, 4°C), remove the supernatant into the injection vial with internal intubation for machine analysis; in addition, each sample was removed and mixed with 20 μL of supernatant for quality control.

### 2.2 Quality control sample preparation

Quality control (QC) samples were prepared by mixing the extraction liquid of all samples in equal volume, the body of each QC is the same as the sample, treated and detected in the same way as the analytical sample, in the course of instrumental analysis, every 5–15 analyses a QC sample is inserted into the sample to examine the stability of the entire testing process.

### 2.3 LC–MS detection

The instrument platform for this LC–MS analysis is the Thermo Feld Ultra High Performance Liquid Chromatography-Tandem Fourier Transform Mass Spectrometry UHPLC-Exploris 240 system.

Chromatographic conditions: The chromatographic column was an ACQUITY UPLC HSS T3 (100 mm × 2.1 mm i.d., 1.8 μm, Waters, Milford, USA); mobile phase A is 95% water +5% acetonitrile (containing 0.1% formic acid), and mobile phase B is 47.5% acetonitrile +47.5% isopropyl alcohol +5% water (containing 0.1% formic acid). The sample size was 3 μL, and the column temperature was 40°C.

Mass spectrometry conditions: Samples were ionized by electrospray, and mass spectrum signals were collected by positive and negative ion scanning modes, respectively. Specific parameters are listed in Table 1.

**Table 1:** Mass spectrum parameter.

Description	Argument
Scan type (m/z)	70–1,050
Sheath gas flow rate (arb)	60
Aux gas flow rate (arb)	20
Heater temp (°C)	350
Capillary temp (°C)	320
Spray voltage (+) (V)	3,400
Spray voltage (−) (V)	−3,000
S-Lens RF level	70
Normalized collision energy (eV)	20,40,60
Resolution (Full MS)	60,000
Resolution (MS2)	15,000

### 2.4 Identification of metabolites

The original data were imported into the metabolomics processing software Progenesis QI (Waters Corporation, Milford, USA). Line filtering, peak identification, integration, retention time correction, peak alignment and so on, and finally the retention time, mass charge ratio and peak intensity are obtained. Data matrix of information. Then the software was used to identify the characteristic peak database, and the MS and MS/MS mass spectrum information was combined with the metabolite number. The MS mass error was set to <10 ppm, and the metabolites were identified according to the secondary mass spectrometry matching score. The main database for http://www.hmdb.ca/, https://metlin.scripps.edu/, and other mainstream public databases and self-created databases.

## 3 Results

### 3.1 RSD diagram of QC sample

**Figure 1:**
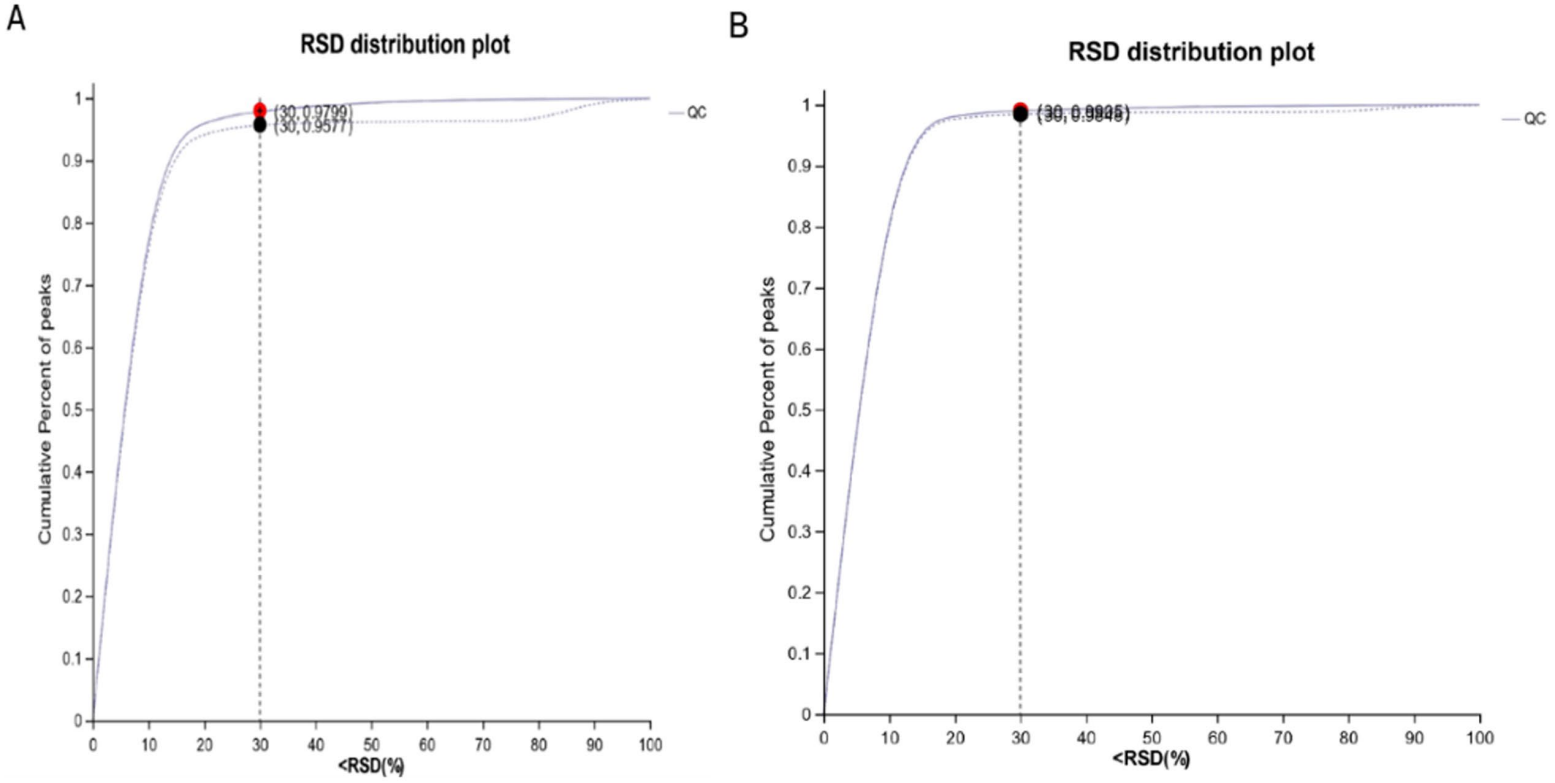
RSD diagram of QC sample. **(A)** RSD diagram of QC sample in cationic mode; **(B)** RSD diagram of QC sample in anionic mode.

By plotting the relative standard deviation (RSD) of positive and negative ions in QC samples, it can be seen from Figures 1A,B that the RSD of positive and negative ions mode is <30%, and the cumulative proportion of peaks is >70%, indicating that the overall data is qualified, and the test results can be used for further analysis.

### 3.2 PCA analysis

**Figure 2:**
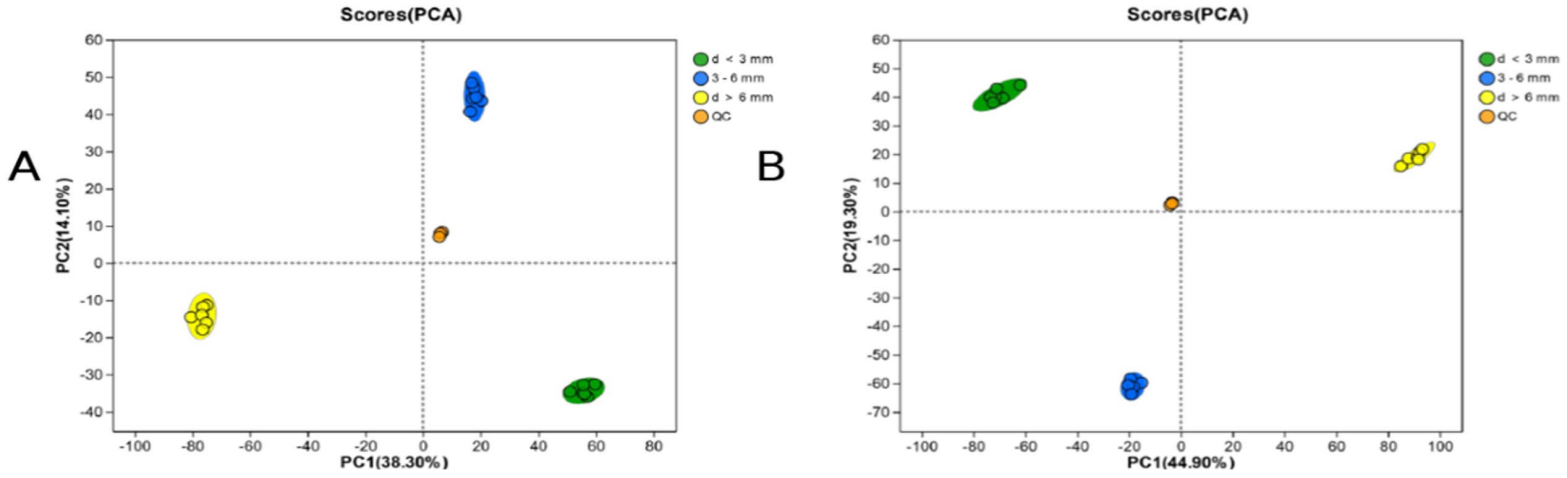
PCA scores for each subgroup. **(A)** PCA scores for each subgroup and the QC sample in ESI (+) mode; **(B)** PCA scores for each subgroup and the QC sample in ESI (−) mode.

PCA analysis can observe the trend of inter-group separation in the experimental model and whether there are any anomalies, and reflect the inter-group and intra-group variability of the original data. As shown in the Figure 2, in the positive ion mode (A) and the negative ion mode (B), R2X (the explanation of the difference in the X variable of the model) is 0.383 and 0.449, respectively. All QC samples are closely clustered, and all samples are within the 95% confidence interval, which proves that the repeatability of this experiment is good, the operation of the instrument and the analysis system are stable, and the data are reliable (Table 2).

**Table 2:** Top 20 differential metabolites in d < 3 mm vs. 3–6 mm.

Metabolite	VIP	P	Ion Mode	FD
5’-N-Methylcarboxamidoadenosine	3.1803	2.23E-06	POS	↓
Lactose	3.0726	1.59E-07	POS	↓
Naringin	2.9207	1.48E-08	POS	↓
PG [20:3(5Z,8Z,11Z)/22:6(5Z,7Z,10Z,13Z,16Z,19Z)-OH(4)]	4.2885	2.03E-08	POS	↓
Soyasaponin aa	4.1951	1.06E-08	POS	↓
3-Nitrotyrosine	3.562	6.13E-10	POS	↓
Penicilloic acid	2.994	9.08E-12	POS	↓
Albafuran C	3.5343	6.98E-12	POS	↓
Paliperidone	3.761	1.07E-17	NEG	↓
5’-N-Ethylcarboxamidoadenosine	2.9225	3.26E-14	POS	↓
Sambunigrin	2.9926	3.82E-06	POS	↑
Salicylamide glucuronide	3.3442	1.54E-08	POS	↑
Leptine I	4.7537	4.12E-09	POS	↑
Methoxyphenanthrene	2.9329	3.42E-14	NEG	↑
Butaprost	3.3437	3.34E-13	NEG	↑
17-phenyl trinor PGF2alpha diethyl amide	2.9893	8.27E-14	POS	↑
(6Z)-11-(3-Pentyloxiran-2-yl) undeca-6,9-dienoylcarnitine	3.3334	2.23E-14	NEG	↑
Quinoxalinol, 3-methyl-, 2-formate	3.0551	7.17E-13	NEG	↑
Tiazofurin	3.3695	1.98E-05	POS	↑
Isopyridoxal	3.1967	2.33E-05	POS	↑

### 3.3 PLS-DA score

**Figure 3:**
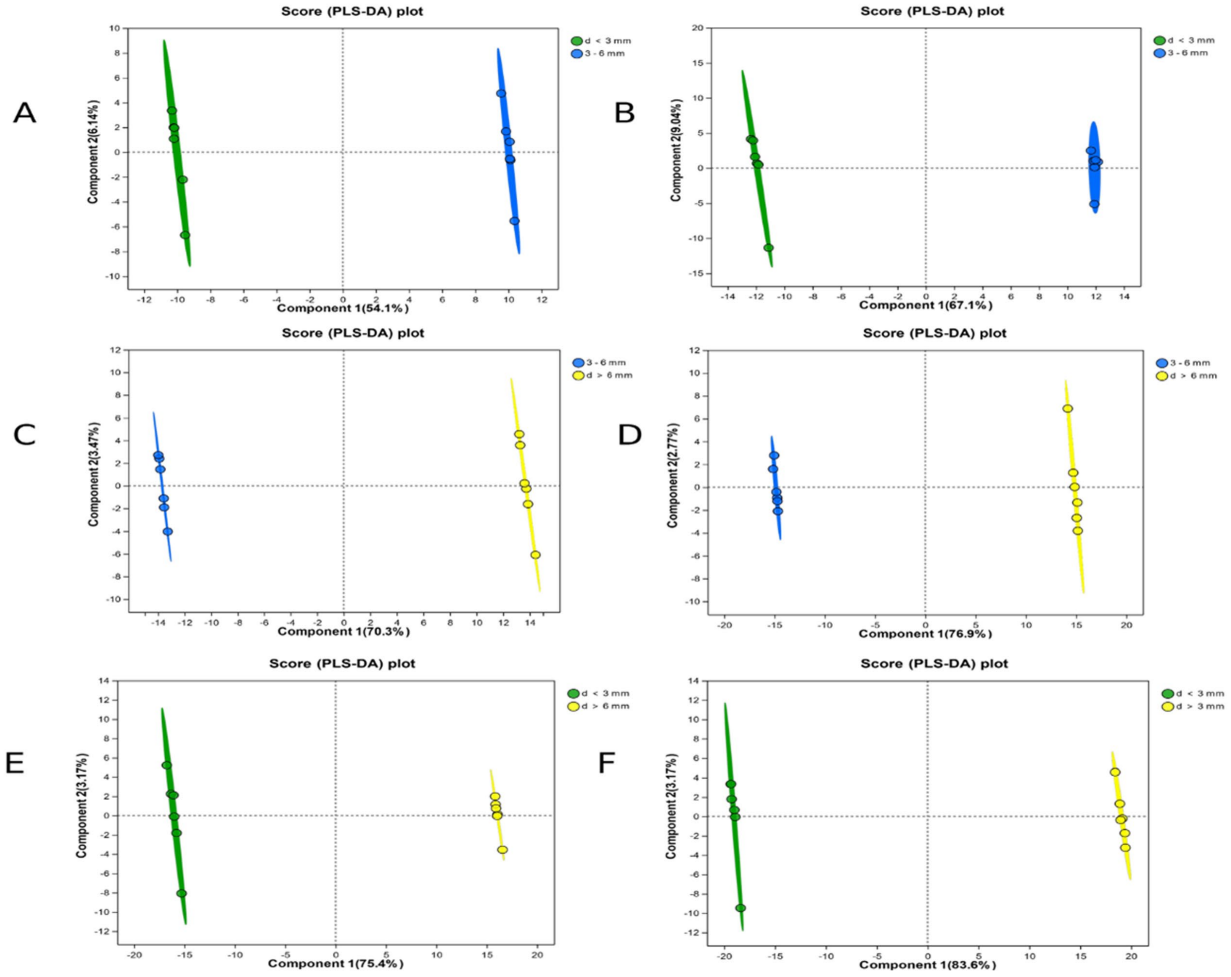
PLS-DA scores for each group in ESI(+) and ESI(−) mode. **(A)** d < 3 mm and 3–6 mm PLS-DA scores in ESI(+) mode; **(B)** d < 3 mm and 3–6 mm PLS-DA scores in ESI(−) mode; **(C)** 3–6 mm and d > 6 mm PLS-DA scores in ESI(+) mode; **(D)** 3–6 mm and d > 6 mm PLS-DA scores in ESI(−) mode; **(E)** d < 3 mm and d > 6 mm PLS-DA scores in ESI(+) mode; **(F)** d < 3 mm and d > 6 mm PLS-DA scores in ESI(−) mode.

PLS-DA score is partial least square discriminant analysis, which is a discriminant analysis method in multivariate data analysis technology. The PLS-DA score map is commonly used to visually show the classification effect of the model, which can eliminate irrelevant systematic errors and improve the difference between the two groups. The greater the degree of separation of the two groups of samples in the differential metabolite map, the more significant the classification effect. As can be seen from the Figure 3, the degree of separation of the samples was >50%, indicating that the classification of the follicles was accurate, similar within the same group, and highly reproducible (Table 3).

**Table 3:** Top 20 differential metabolites in 3–6 mm vs. d > 6 mm.

Metabolite	VIP	P	Ion Mode	FD
5-Phenyl-1,3-oxazinane-2,4-dione	3.039	8.61E-09	POS	↑
Phenethylamine glucuronide	3.3416	2.08E-07	POS	↑
Oxidized glutathione	3.2614	2.16E-08	NEG	↑
Glycine, N-[1-(phenylacetyl)-L-prolyl]-	2.9554	8.77E-15	NEG	↑
Imazapyr	3.1568	3.39E-17	POS	↑
Methionyl-Lysine	3.5508	3.31E-21	POS	↑
Penicilloic acid	3.4544	3.31E-21	POS	↑
Ecgonine Ethyl Ester	3.249	3.31E-21	POS	↑
PG [18:1(12Z)-2OH(9,10)/22:4(7Z,10Z,13Z,16Z)]	3.2562	3.31E-21	POS	↑
Paliperidone	3.098	3.31E-21	NEG	↑
Arabinosylhypoxanthine	3.2406	3.31E-21	POS	↑
DIMBOA trihexose	3.0215	3.31E-21	NEG	↑
Rifampicin	4.0833	3.31E-21	POS	↓
Blumenol C O-[rhamnosyl-(1- > 6)-glucoside]	3.0586	3.31E-21	POS	↓
Phenylalanyltryptophan	3.6988	3.31E-21	NEG	↓
1-Methylhistidine	3.4279	3.31E-21	POS	↓
PS [TXB2/18:3(6Z,9Z,12Z)]	3.0486	3.31E-21	NEG	↓
Leptine I	4.1384	3.31E-21	POS	↓
Phe Trp	3.7759	3.31E-21	POS	↓
Ggstop	3.5324	3.31E-21	POS	↑

### 3.4 Differential metabolite screening

**Figure 4:**
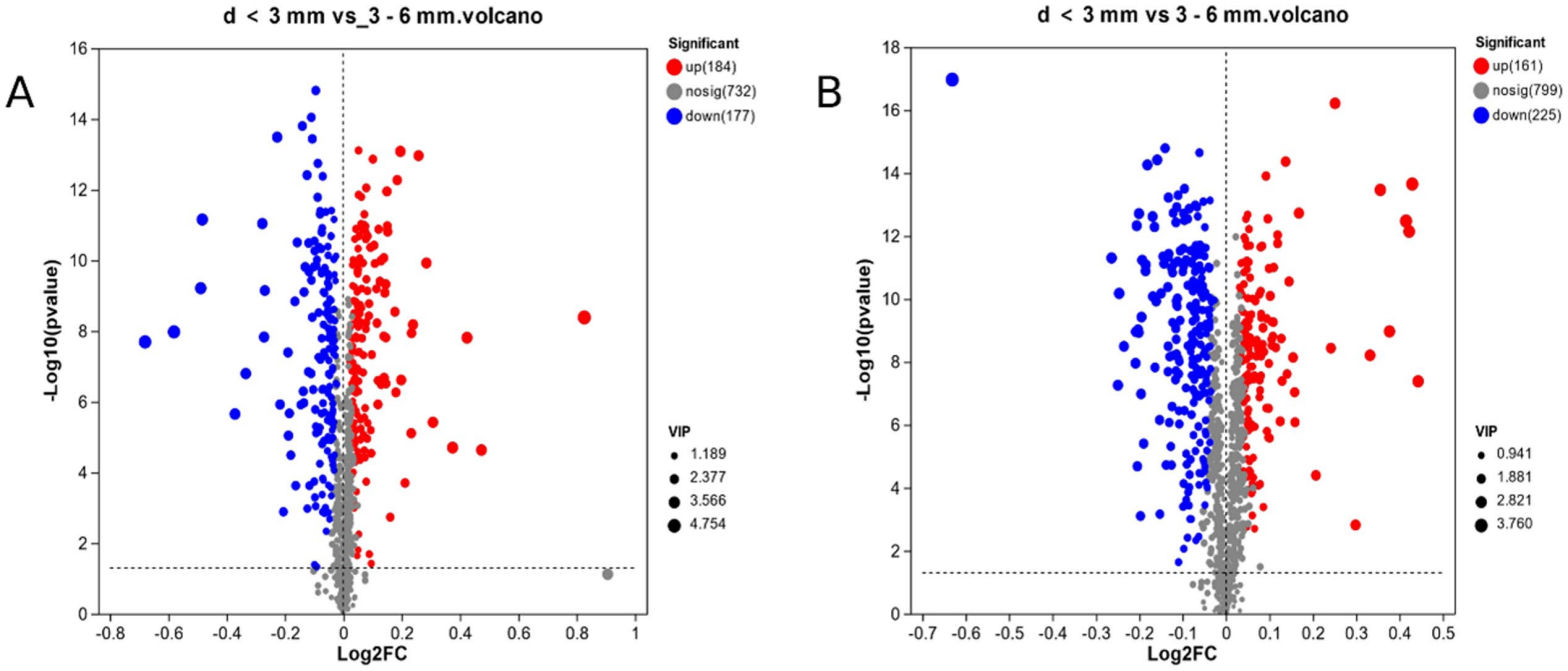
d < 3 mm vs. 3–6 mm metabolic sets in ESI(+) and ESI(−) mode. **(A)** d < 3 mm vs. 3–6 mm metabolic sets in ESI(+) mode; **(B)** d < 3 mm vs. 3–6 mm metabolic sets in ESI(−) mode.

In this experiment, the PLS-DA model was used to calculate the projected importance of variables (VIP) and the *T*-test was used to screen the differential metabolites. By setting VIP > 1, *p* < 0.05, in Figure 4A, a total of 361 differential metabolites were screened in d < 3 mm and 3–6 mm under ESI (+) mode. Compared to 3–6 mm, 184 metabolites were up-regulated and 177 metabolites were down-regulated in d < 3 mm. In Figure 4B, a total of 386 different metabolites were screened in ESI (−) mode, of which 161 metabolites were up-regulated and 225 metabolites were down-regulated in the 3–6 mm range, d < 3 mm.

**Figure 5:**
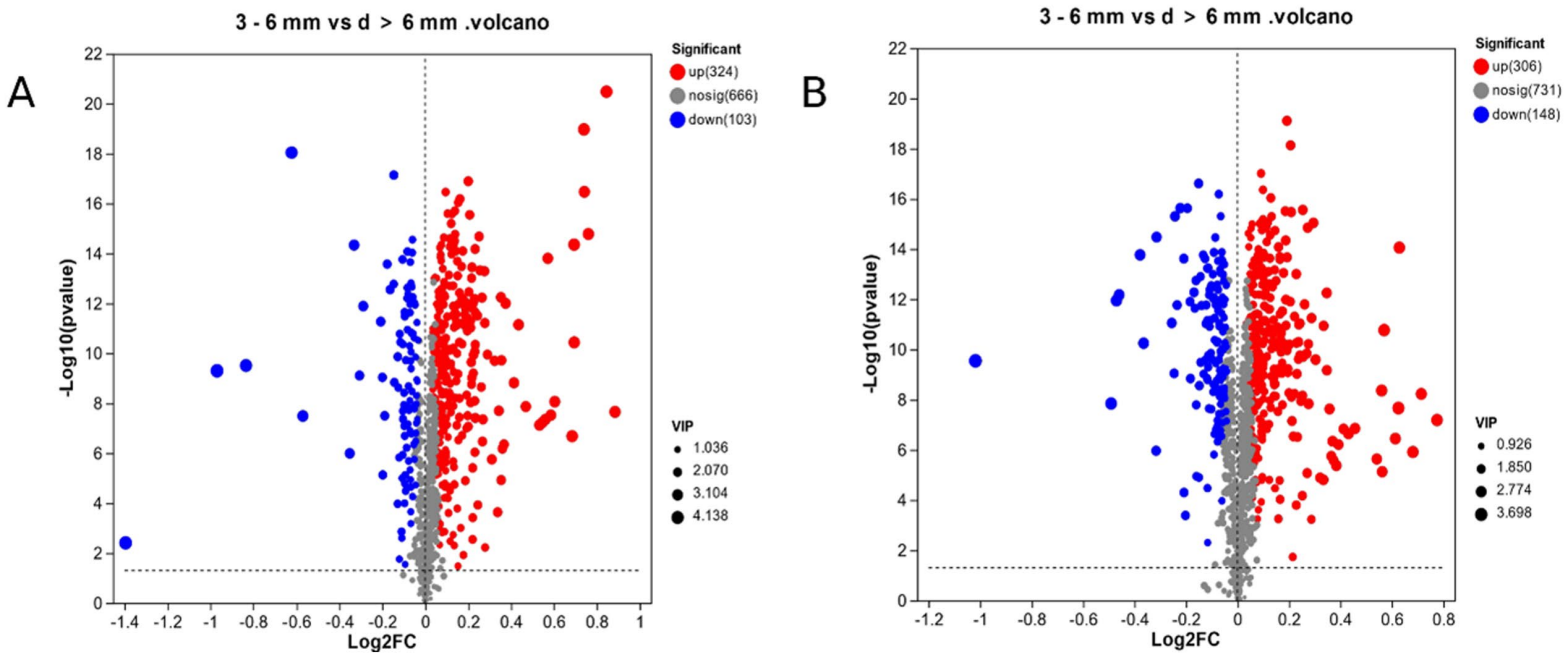
3–6 mm vs. d > 6 mm metabolic sets in ESI(+) and ESI(−) mode. **(A)** 3–6 mm vs. d > 6 mm metabolic sets in ESI(+) mode; **(B)** 3–6 mm vs. d > 6 mm metabolic sets in ESI(−) mode.

In ESI (+) mode, in Figure 5A, a total of 427 different metabolites were screened in 3–6 mm and d > 6 mm, compared to d > 6 mm, in 3–6 mm, 324 metabolites were up-regulated and 103 metabolites were down-regulated. In Figure 5B, a total of 454 different metabolites were screened in ESI (−) mode, of which 306 metabolites were up-regulated and 148 metabolites were down-regulated in 3–6 mm compared to d > 6 mm.

### 3.5 Differential metabolite VIP value

In Tables 2, 3, by setting VIP > 1 and *p* < 0.05, the top 20 differential metabolites in d < 3 mm vs. 3–6 mm and 3–6 mm vs. d > 6 mm were screened.

### 3.6 Venn diagram

**Figure 6:**
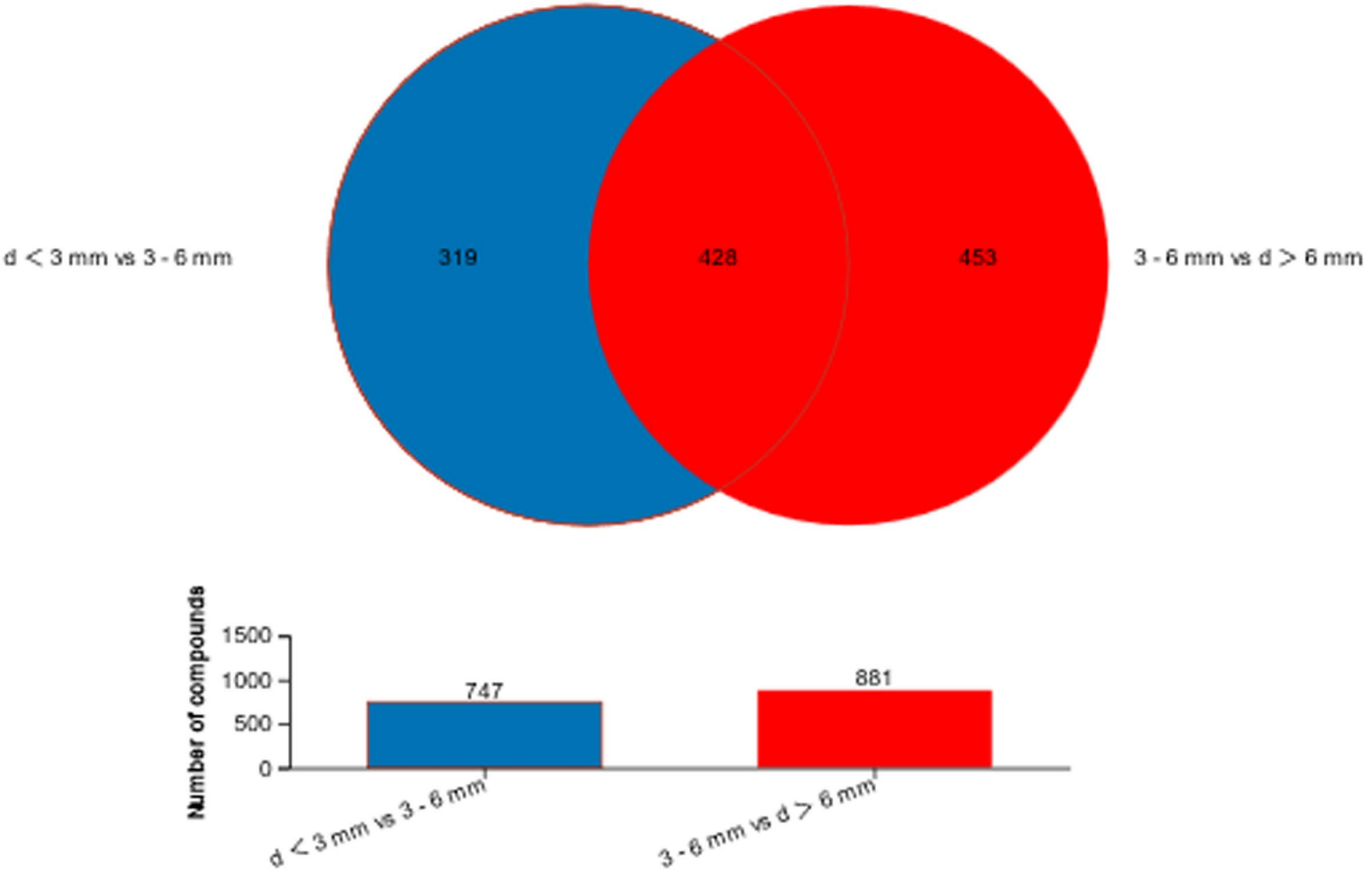
Venn diagram of d < 3 mm vs. 3–6 mm metabolic set vs. d > 6 mm vs. 3–6 mm metabolic set.

The different metabolites in the two comparison groups were collected and combined, and a Venn diagram (Figure 6) was constructed for the metabolic set to show the common and unique metabolites of each metabolic set. The overlapping part represented the number of metabolites shared by multiple metabolic sets, while the non-overlapping part represented the number of metabolites unique to the metabolic set, and the number represented the corresponding metabolite number. The bar graph shows the number of metabolites contained in each metabolic set. In Figure 6, a total of 747 differential metabolites were found in d < 3 mm and 3–6 mm, and a total of 881 differential metabolites were found in 3–6 mm and d > 6 mm, and by comparing these two groups of differential metabolites, 428 differential metabolites were found together.

### 3.7 Cluster analysis of metabolites

**Figure 7:**
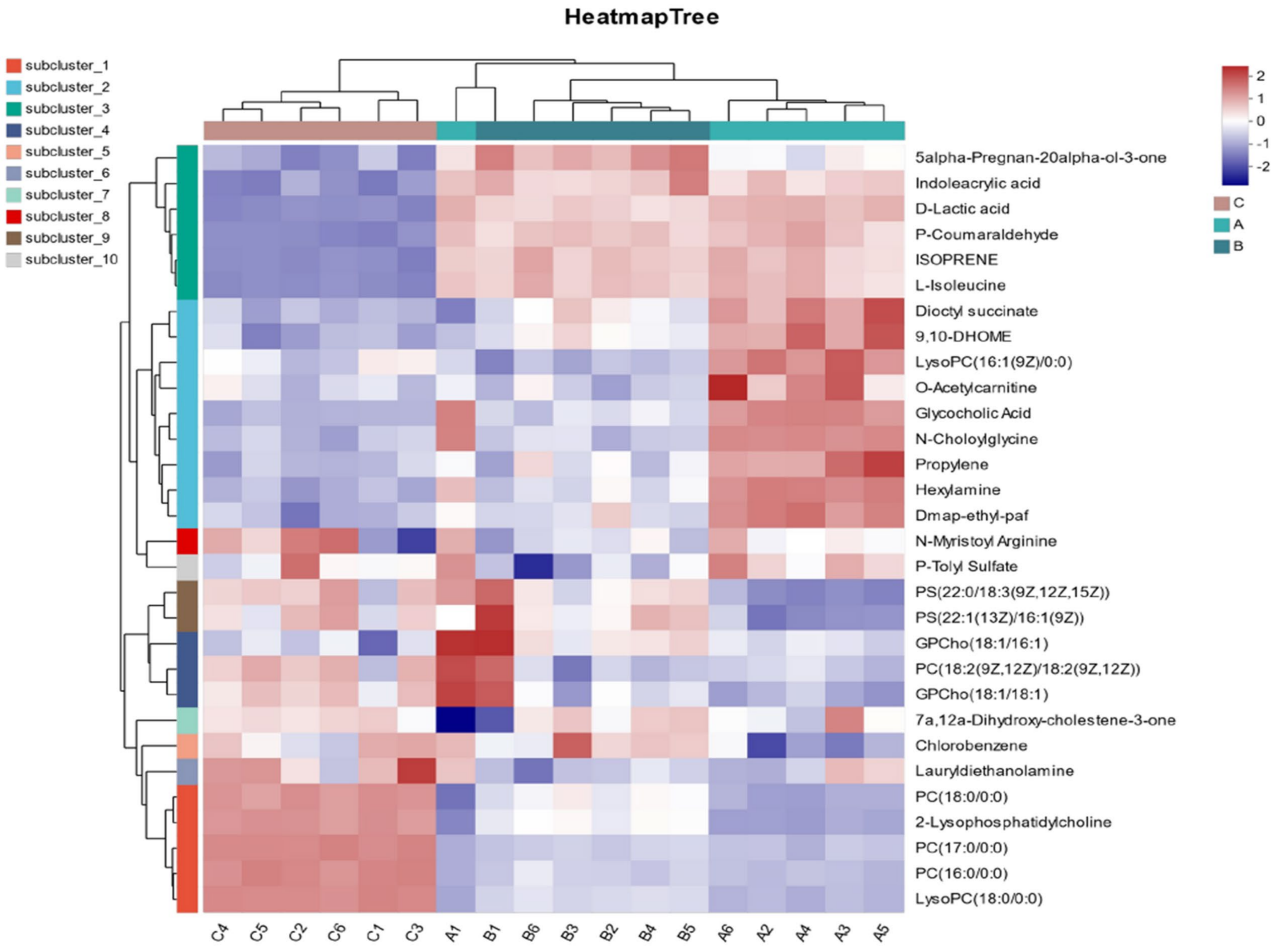
Metabolite cluster analysis. A represents follicle d < 3 mm. B represents 3–6 mm, C represents d > 6 mm.

In Figure 7, through the cluster analysis of differential metabolites, the expression of different metabolites in different samples can be intuitively expressed, as shown in the figure. It can be seen from the figure that all samples can be separated, and the differential metabolites in the same group have similar expression patterns, indicating that the classification of follicles is accurate and there is no difference. As follicle diameter increased, 2-Lysophosphatidylcholine, PC (17:0/0:0), PC (16:0/0:0), and LysoPC (18:0/0:0) were down-regulated; Dioctyl succinate, P-Coumaraldehyde, ISOPRENE, L-Isoleucine, Dioctyl succinate up-regulated.

### 3.8 Differential metabolite KEGG pathway enrichment

**Figure 8:**
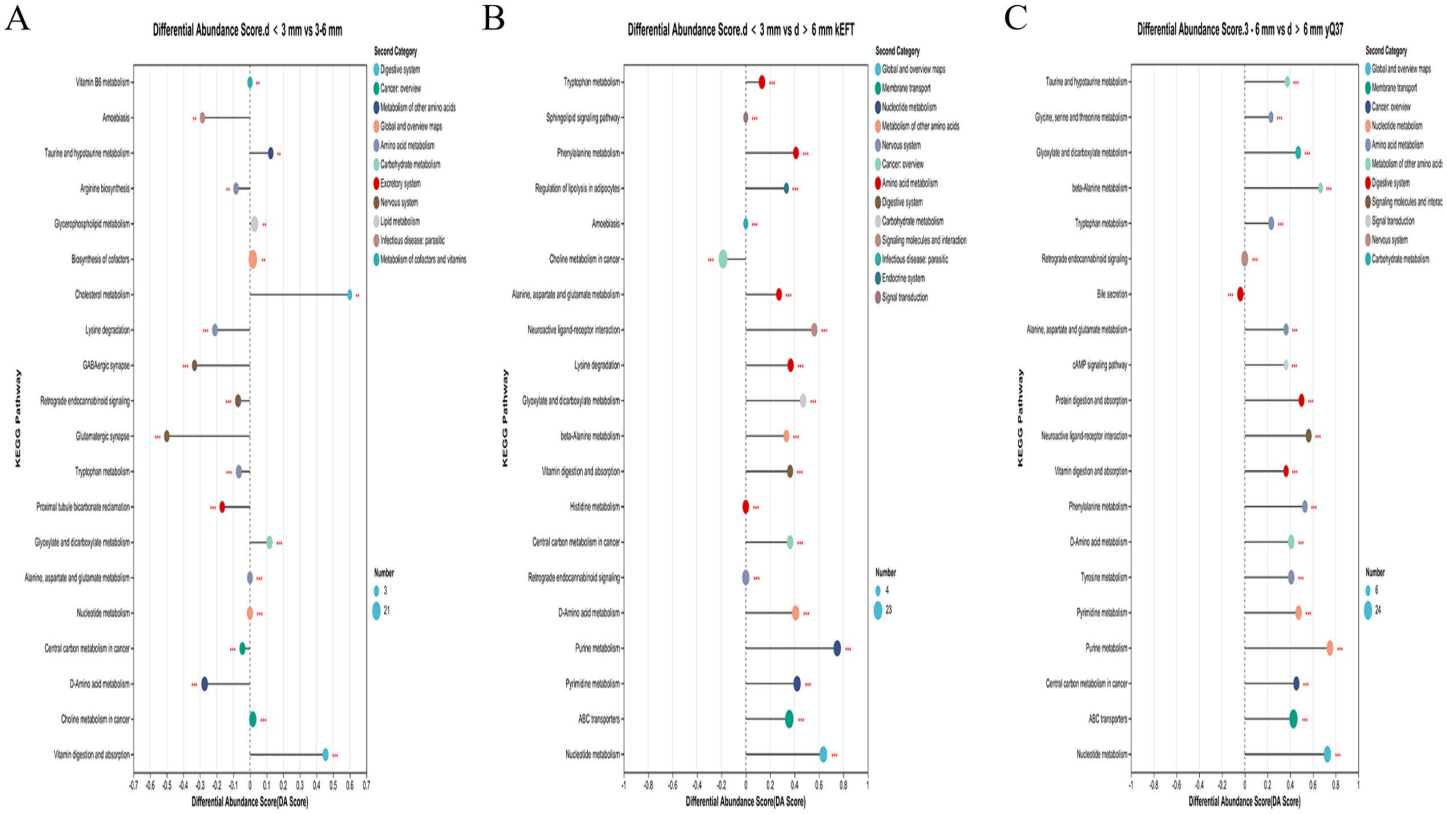
Enrichment of KEGG pathway of different metabolites in follicles. **(A)** Enrichment of KEGG pathway of different metabolites in follicles with d < 3 mm and 3–6 mm; **(B)** KEGG pathway enrichment diagram of 3–6 mm and d > 6 mm differences; **(C)** KEGG pathway enrichment diagram of different metabolites with d < 3 mm and d > 6 mm.

Enrichment analysis of the differential metabolites of d < 3 mm and 3–6 mm and 3–6 mm and d > 6 mm was performed. The hypergeometric distribution algorithm was used to obtain the path of significant enrichment of metabolites in the metabolic concentration. The BH method was used to correct the *p*-value. Screening for significant enrichment pathways. In Figure 8A, it was found that the different metabolites in follicles with d < 3 mm and 3–6 mm were mainly enriched in vitamin B6 metabolism, glycerol-phospholipid metabolism, cofactor biosynthesis, choline metabolism, amino acid metabolism and nucleotide metabolism. In Figure 8B, different metabolites in follicles of 3–6 mm and d > 6 mm were mainly concentrated in taurine metabolism, amino acid metabolism, nucleotide metabolism, choline metabolism, vitamin digestion and absorption, bile secretion and ABC transporter. In Figure 8C, different metabolites in follicles with d < 3 mm and d > 6 mm were mainly concentrated in amino acid metabolism, nucleotide metabolism, choline metabolism, ABC transporter and adipocyte lipolysis.

### 3.9 Classification of KEGG compounds

**Figure 9:**
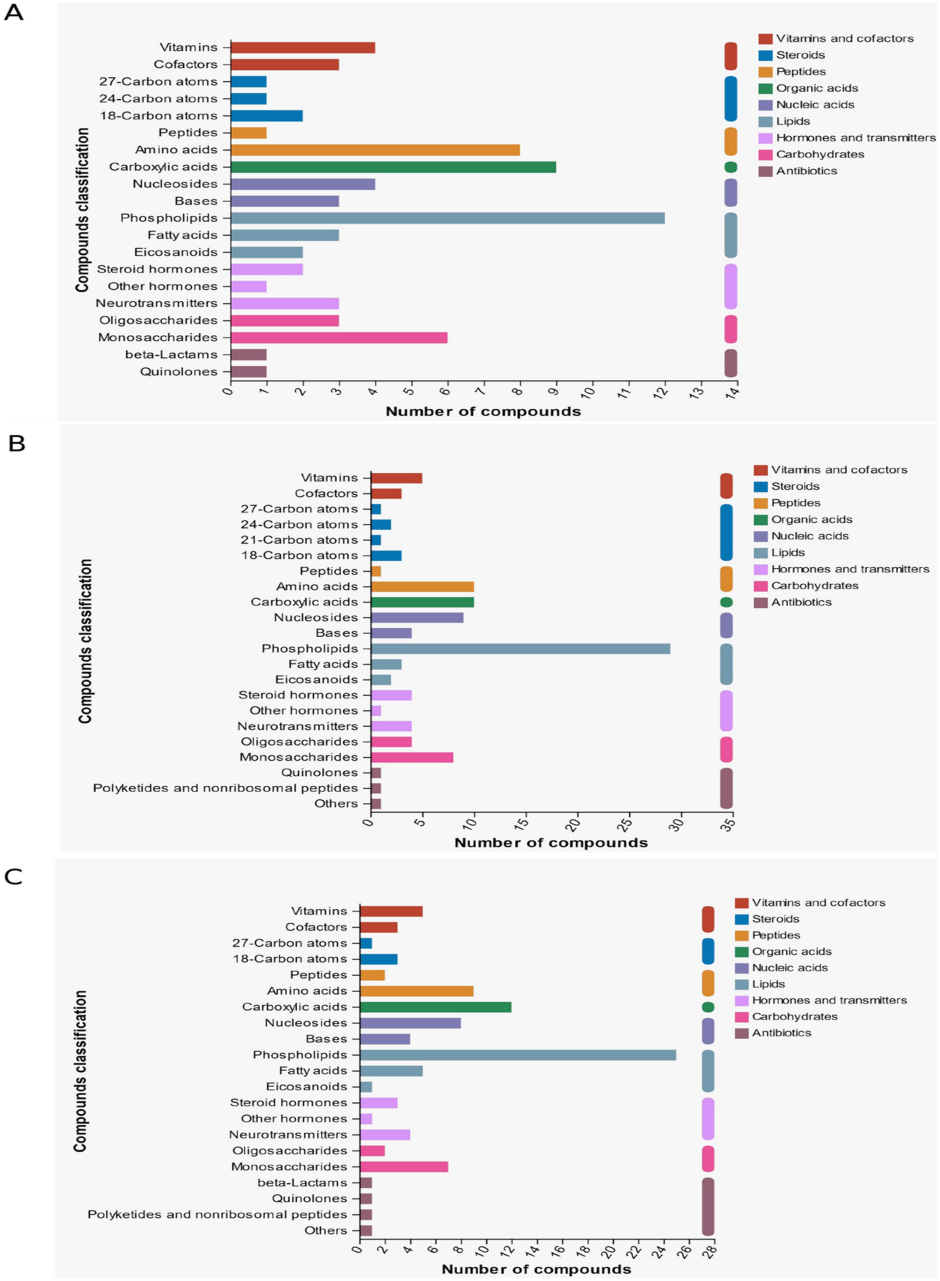
KEGG compound classification. **(A)** d < 3 mm vs. 3–6 mm KEGG compound classification. **(B)** 3–6 mm vs. d > 6 mm KEGG compound classification. **(C)** d < 3 mm vs. d > 6 mm KEGG compound classification.

By KEGG classification of compounds in the follicular fluid, as shown in Figure 9A, the highest percentage of compounds in d < 3 mm vs. 3–6 mm were Phospholipids, Carboxylic acids and Amino acids, whereas in Figures 9B,C, the highest percentage of compounds in 3–6 mm vs. d > 6 mm as well as in d < 3 mm and d > 6 mm were all Phospholipids.

### 3.10 Classification of HMDB compounds

**Figure 10:**
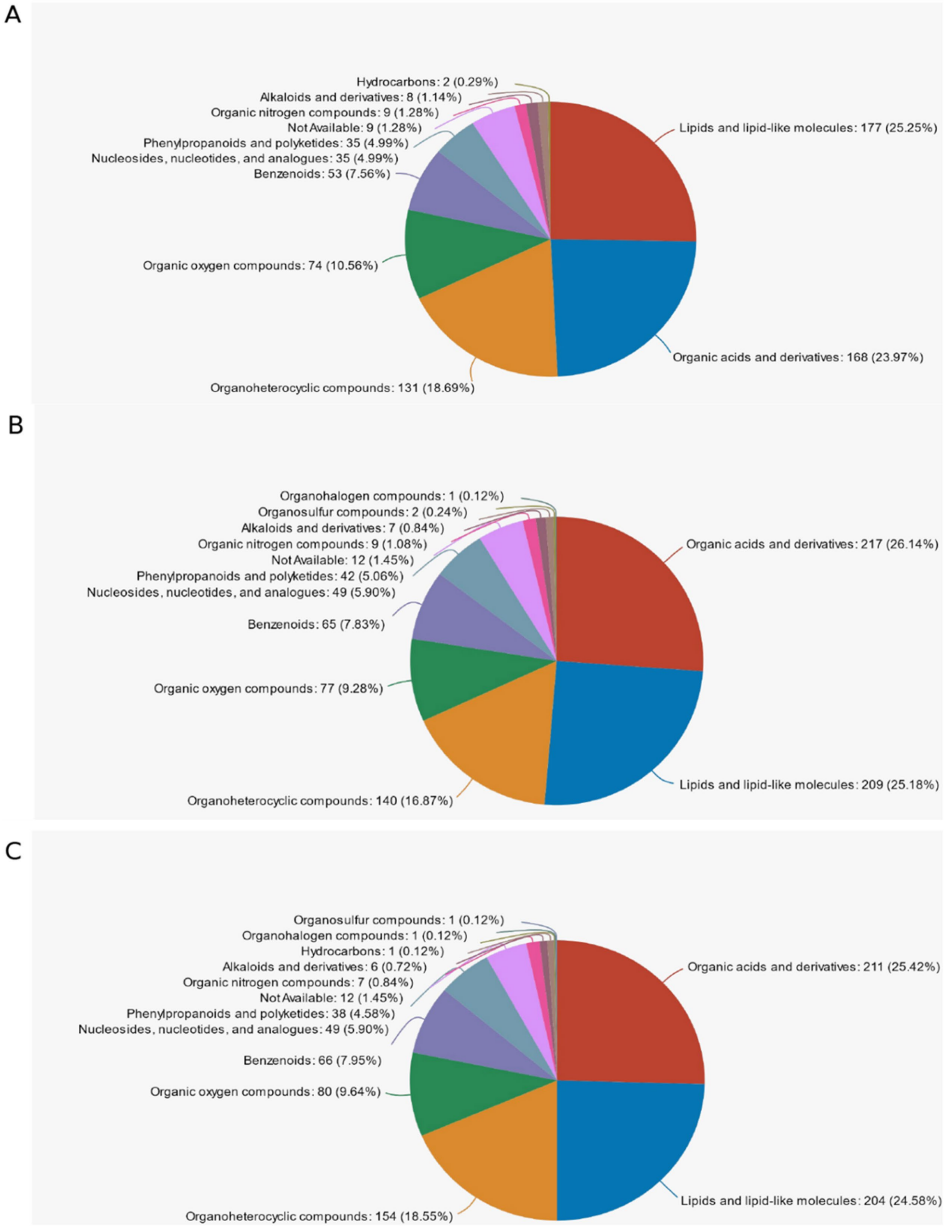
Classification of HMDB compounds. **(A)** d < 3 mm vs. 3–6 mm classification of HMDB compounds; **(B)** 3–6 mm vs. d > 6 mm classification of HMDB compounds; **(C)** d < 3 mm vs. d > 6 mm classification of HMDB compounds.

The name of the selected HMDB tier (Superclass, Class, or Subclass) and the percentage of metabolites accounted for are shown in descending order according to the number of metabolites. Different colors in each pie chart in the figure represent different HMDB classifications, and their area indicates the relative percentage of metabolites in that classification. In Figure 10A, in d < 3 mm and 3–6 mm, the highest percentage was Lipid and Lipid-like molecules at 25.25%, the second was Organic acids and derivatives at 23.97% and the third was Organopheterocyclic compounds at 18.69%. In Figure 10B, among 3–6 mm and d > 6 mm, the highest percentage was Organic acids and derivatives at 26.14%, the second was Lipid and Lipid-like molecules at 25.18%, and the third was Organpheterocyclic componds at 16.87%. In Figure 10C, among the d < 3 mm and d > 6 mm, the highest percentage was Organic acids and derivatives at 25.42%, the second was Lipid and Lipid-like molecules with 24.58%, and the third was Organpheterocyclic componds with 18.55%.

### 3.11 Purine metabolism

**Figure 11:**
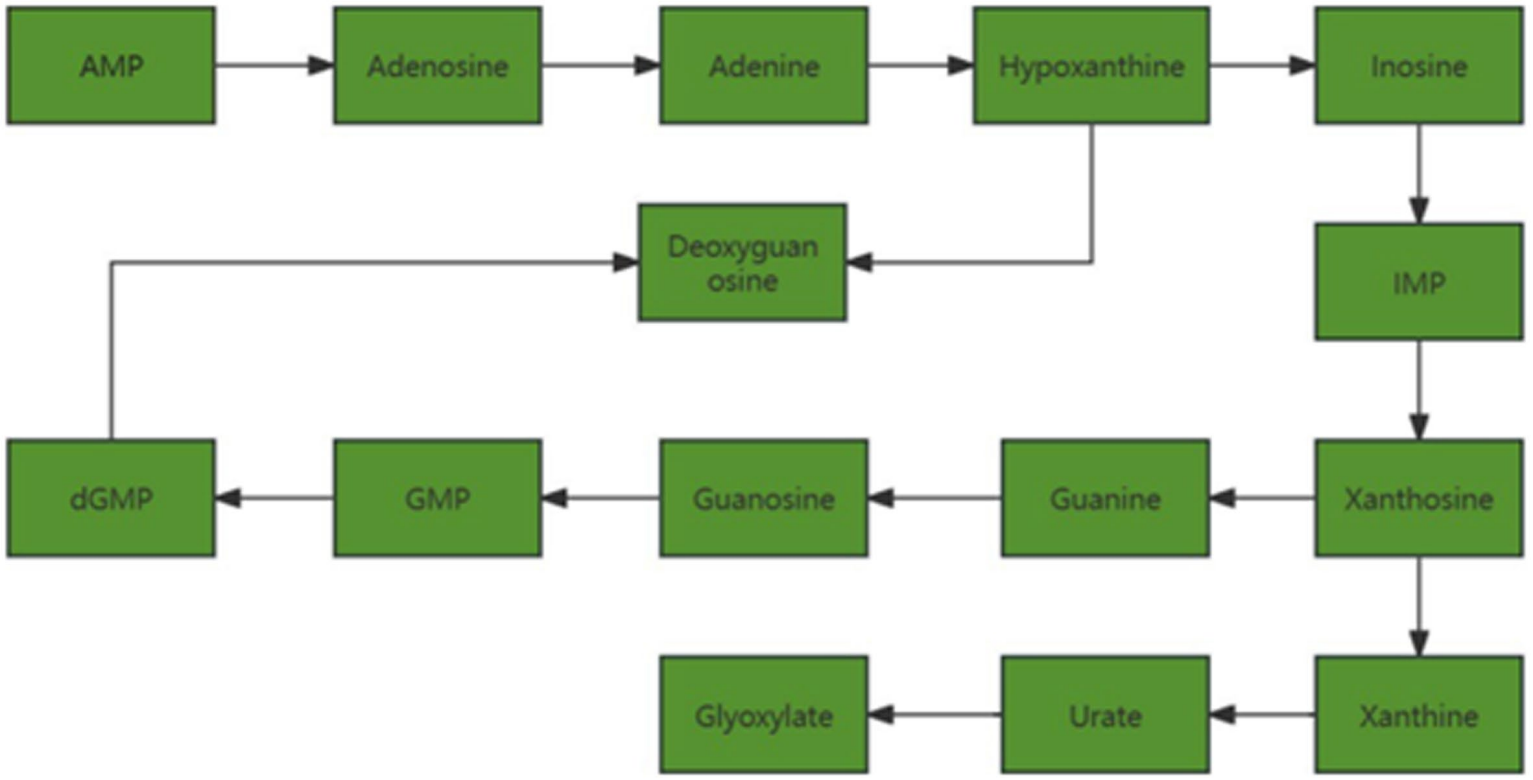
Changes in pathways related to purine metabolism.

As shown in Figure 11, with the increase of follicle diameter, metabolites related to purine metabolism in follicular fluid are down-regulated.

### 3.12 Pyrimidine metabolism

**Figure 12:**
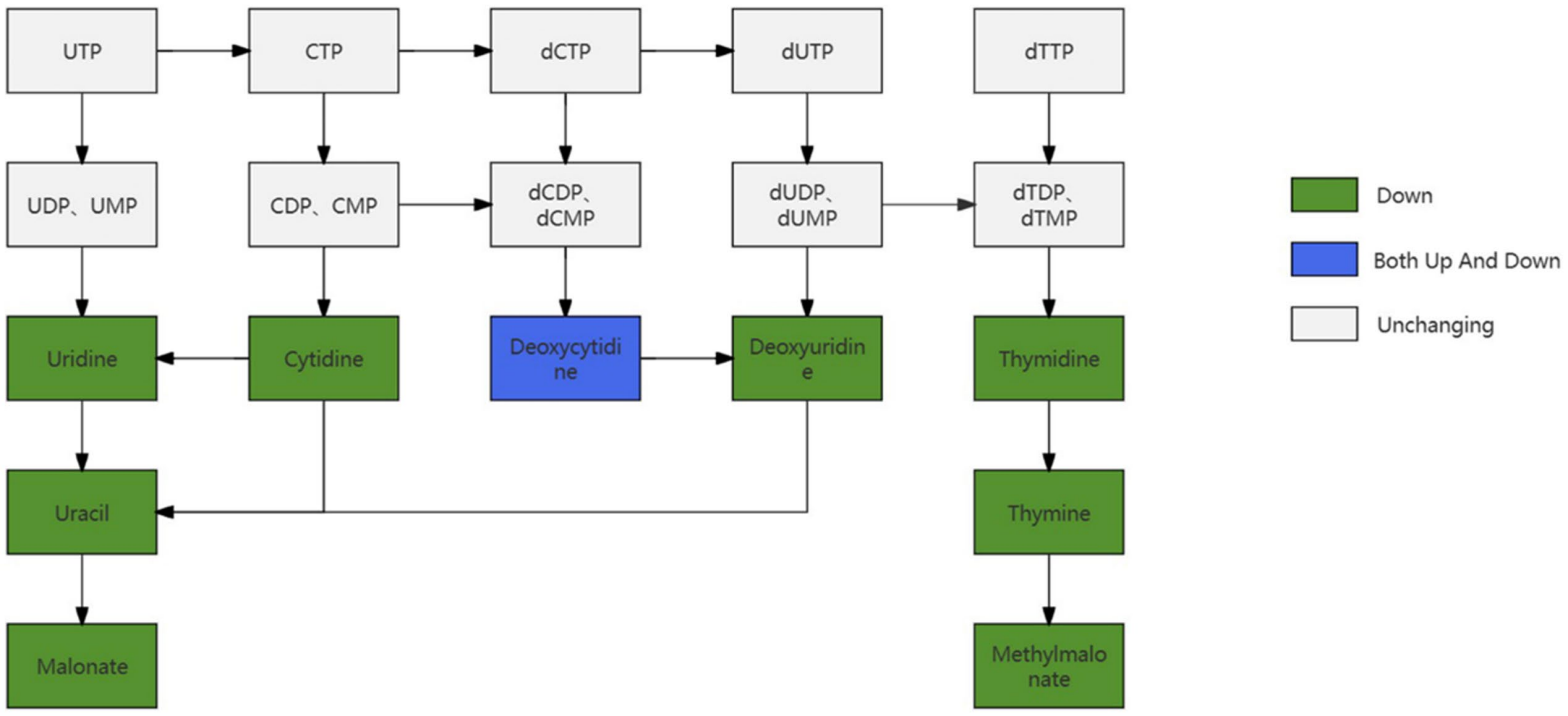
Changes in pathways related to pyrimidine metabolism.

By examining the metabolites in follicles of different sizes and comparing them to the pyrimidine metabolism-related pathway, it was found that most of the metabolites in the pyrimidine metabolism-related pathway appeared to be down-regulated, and some of the metabolites remained unchanged, whereas there was both up-regulation as well as down-regulation of Doxycytidine in the pyrimidine metabolism pathway as illustrated in Figure 12.

## 4 Discussion

In this experiment, using LC–MS detection of follicular fluid of different sizes from yaks, it was found that the differential metabolites in follicular fluid of d < 3 mm and 3–6 mm and in follicular fluid of 3–6 mm and d > 6 mm were mainly concentrated in amino acid metabolism, this is also consistent with previous literature (18). Studies have shown amino acids can be converted into a variety of nitrogenous physiologically active substances such as purines, pyrimidines and catecholamines, while the excess amino acids were used for dechomination to provide energy. In the process of amino acid metabolism, amino acids were first dehydrogenated, which were then hydrolysed to *α*-ketoacids and NH3. In addition, amino acids transfer the amino group of one amino acid to another amino acid under the action of aminotransferase; expect lysine, proline and hydroxyproline, most amino acids participate in transamination (19). Although transamination is common in the body, the majority of amino acid deamination in the body is through a combined transamination transfer of amino acid to α-ketoglutaric acid to produce α-ketoic acid and L-glutamic acid, by oxidative deamination to produce ammonia and α-ketoglutaric acid, these were metabolized by amino acids to produce alpha-ketoacids which can be further converted to sugars and lipids (20–22). Glutamate has a very important role in follicular development, glutamate is the main neurotransmitter that stimulates neuroendocrine activity during mammalian reproduction, GnRH release is stimulated by glutamate, and, glutamate activity is regulated by intrafollicular oestradiol and progesterone, resulting in the regulation of follicular development (23). In addition, aromatic amino acids convert phenylalanine to tyrosine, which can be converted to dopamine and then to norepinephrine, epinephrine, thyroid hormone, melanin, and so on. Tryptophan was broken down into pyruvate and acetoacetic acid and was used in the synthesis of niacin and vitamin B5 (24, 25). Previous studies have also shown that folic acid can improve the ovarian environment, preventing oxidative stress and increasing cell viability in ovarian cells (26, 27). Folic acid administered intramuscularly can affect energy efficiency and promote the development of dominant follicles, but it is not known whether folic acid acts directly on follicles and the hypothalamic–pituitary-ovarian axis (28). Among the sulphur-containing amino acids, cysteine was involved in the synthesis of glutathione to play an antioxidant role, while methionine was an important methyl supplier in the body, involved in the synthesis of adrenaline, choline, carnitine, and so on. In addition to this, additional reports have found that leucine and isoleucine in the follicle show a positive correlation on the development and implantation capacity of the oocyte at the completion of follicular development and at later stages of the oocyte’s development (29), this is also consistent with our screening of isoleucine as a key up-regulated metabolite. Moreover, leucine and isoleucine are branched-chain amino acids that can be broken down by mitochondria in the follicle to provide ATP (30). Whereas the metabolism of these amino acids plays a crucial role in the quality of the subsequent oocyte as well as the embryo (31), glycine and alanine are important indicators of post-fertilization oocytes (32). Amino acids play a variety of key roles in follicular development, includes synthesis of proteins required for follicular development, energy requirements, regulation of follicular osmolality and functioning as a cellular buffer (33).

In addition to this, the development of oocytes within the follicle is regulated by other factors, during *in vivo* maturation, granulosa cells, cumulus cells and other somatic cells secreted various metabolites to modulate oocyte maturation (34, 35). Granulosa cells were the major somatic cells in the follicle. In mammalian ovaries, granulosa cells proliferated first, and when the number of proliferations reached a certain level, oocytes began to develop. Oocytes were the regulatory object of granulosa cells; importantly, however, granulosa cells can be regulated by oocytes. The majority of several currently understood paracrine signaling molecules were secreted by oocytes, the most important being growth differentiation factors (GDF9) and bone morphogenetic proteins (BMPs) (36). BMP15 and GDF9 had many functions, not only regulating oocyte development, but also regulating intercellular communication, resisting granulosa cell apoptosis, hormone secretion and protein expression (37–39). Our KEGG pathway enrichment results identified a number of pathways that may be relevant to follicular development and oocyte maturation, including the cAMP pathway, which is important for the ability of oocytes to undergo normal meiosis. During oocyte development, oocytes were regulated by gap junction transport substances. Granular cells can supply and transport small molecules of nutrients, vitamins, calcium ions, metabolic precursors and signaling molecules needed by oocytes through gap junctions. In the process of meiosis, oocytes have to be blocked in the middle of meiosis II and wait for fertilization. Granulosa cells can release large amounts of cAMP, reduce the activity of cell cycle-dependent enzymes (CDKs) and arrest meiosis (40, 41). During preovulation, oocytes were regulated by high levels of LH, which can activate the EGFR pathway, reduce cGMP levels, activate (PDE) 3A to hydrolyse cAMP and restore oocyte meiosis (42).

Our study identified the presence of ABC transporter proteins in ovarian follicles, which is also consistent with the literature that ABC transporter proteins were first detected in goat ovarian follicles in 2018 (43). And that the main role of these transporter proteins is to transport molecules such as sugars, amino acids, nucleotides, and lipids across the biofilm using the energy generated by the hydrolysis of ATP, and that the expression of ABC transporter proteins in granulosa cells may be associated with an increase in steroids. Granulosa cells produce pyruvate and lactic acid by glycolysis. In addition to this, our study revealed that many energy acquisition pathways are enriched in follicular fluid. Granulosa cells obtain energy mainly through glycolysis, whereas oocytes rely mainly on mitochondrial oxidative phosphorylation for energy. Granulosa cells produce pyruvate and lactic acid by glycolysis. These accumulated lactic acid and pyruvate enter the oocyte through the intercellular space and become raw materials for oxidative phosphorylation of oocyte mitochondria (44, 45). The ATP produced by mitochondrial oxidative phosphorylation and TCA in one cycle is approximately 18 times than glycolysis, which can provide sufficient energy for oocyte growth (46). Furthermore, this process is also regulated by FSH, which can promote glycolysis through the AMPK-SIRT1 pathway and enable granulosa cells to take up glucose (47). FSH and LH are the major protein hormones that regulate follicular growth and development. When they bind to receptors, they activate cAMP-dependent physiological processes and promote the activity of steroid hormone-producing enzymes in granulocytes and parietal cells (48). LH and FSH can also regulate follicle growth by regulating these receptors. In addition to steroid hormones, follicles can also synthesize relaxin, inhibin, oxytocin, activin, etc. There are many regulatory factors in the follicles,but most of the factors have an inhibitory effect on follicle growth. These factors can be secreted by dominant follicles and inhibit the development of other follicles.

For the key metabolites we screened for down-regulation, lysophosphatidylcholine can be converted to lysophosphatidic acid by lysophospholipase D (LysoPLD), which is involved in several metabolic pathways as a biologically active lipid mediator (49). Its main functions include induction of platelet aggregation, smooth muscle contraction, stimulation of cell proliferation and chemotaxis (50). As the small follicle develops into a large follicle, the granulosa cells within the follicle need to proliferate and secrete oestradiol, which plays a role in promoting the development of the follicle as well as the maturation of the oocyte. Interestingly, in our study, we found that 2-Lysophosphatidylcholine, PC (17:0/0:0), PC (16:0/0:0), and LysoPC (18:0/0:0) were down-regulated, which may suggest that during follicular development, granulosa cell proliferation occurs mainly during small to medium-sized follicles. In the large follicles, the proliferating granulosa cells mainly secrete oestradiol and progesterone, which is consistent with previous studies that the levels of oestradiol and progesterone are higher in the large follicles than in the small follicles (32). As for the key metabolites we screened for up-regulation, of which leucine has already been described in the previous section, isoprene belongs to the terpenoid group and is a component of steroid hormones, which is constantly used to synthesize oestradiol during the development of small follicles into large follicles to stimulate the maturation of the oocyte (51). There is no literature on the role of dioctyl succinate, P-Coumaraldehyde, in animal reproduction and follicular development. However, follicle development is regulated by a variety of metabolic pathways as well as metabolites, and more information on the mechanisms by which metabolites regulate follicle development needs to be explored further.

## 5 Conclusion

In summary, our study showed that d < 3 mm and 3–6 mm, metabolites were mainly enriched in cofactor biosynthesis, glycine and dicarboxylic acid metabolism, vitamin digestion and absorption, taurine metabolism, nucleotide metabolism, alanine, aspartate, and glutamate metabolism. At 3–6 mm and d > 6 mm, metabolites were mainly enriched in the pathways of nucleotide metabolism, ABC transporter proteins, cAMP signaling pathway, vitamin digestion and absorption, taurine metabolism, alanine, aspartic acid, glutamate metabolism, and purine metabolism. And as follicle diameter increased, the pathways of taurine metabolism, vitamin digestion and absorption, alanine, aspartic acid, and glycine metabolism were all up-regulated.

## Data Availability

The original contributions presented in the study are included in the article/supplementary material, further inquiries can be directed to the corresponding author.
